# Association Between the Systemic Immune-Inflammation Index (SII) and the Severity of Coronary Artery Stenosis Based on the Gensini Score in Patients With Chronic Coronary Syndrome Undergoing Coronary Angiography

**DOI:** 10.7759/cureus.115738

**Published:** 2026-09-03

**Authors:** Stefanus Surya, Eka Ginanjar, Sally A Nasution, Alvina Widhani, Mohamad S Azizi, Mira Yulianti, Hamzah Shatri

**Affiliations:** 1 Cardiology, Cipto Mangunkusumo National General Hospital, Jakarta, IDN; 2 Allergy and Immunology, Cipto Mangunkusumo National General Hospital, Jakarta, IDN; 3 Pulmonology, Cipto Mangunkusumo National General Hospital, Jakarta, IDN; 4 Internal Medicine, Cipto Mangunkusumo National General Hospital, Jakarta, IDN

**Keywords:** coronary artery disease, diagnostic, gensini, inflammation, stenosis

## Abstract

Background

Chronic coronary syndrome (CCS) is characterized by a dynamic course of atherosclerosis and changes in coronary artery function, shaped by lifestyle, exposure to risk factors, pharmacologic interventions, and revascularization, leading to regression, stability, or progression. Inflammatory and immune processes are integral to CCS pathogenesis and are typically chronic and low-grade. The systemic immune-inflammation index (SII = platelet × neutrophil/lymphocyte) reflects systemic inflammatory status, while coronary disease severity is quantified by the Gensini score, an angiographic index that weights percent luminal narrowing by lesion location and collateral significance and sums across segments to represent overall anatomic burden.

Methodology

We conducted a cross-sectional study of adult patients with CCS undergoing elective coronary angiography at Cipto Mangunkusumo National General Hospital from January 2023. Severe coronary stenosis was defined as a Gensini score ≥20. The SII was calculated from complete blood count results obtained within 24 hours before angiography. Discriminative performance of SII was assessed using receiver operating characteristic (ROC) curve analysis, with the optimal cut-off determined by the Youden index. Associations between SII and severe stenosis were quantified as prevalence ratios (PRs) using log-binomial regression models, adjusted for demographic and cardiometabolic covariates.

Results

A total of 296 patients were enrolled. Severe stenosis was identified in 46.3% of patients. The median SII was significantly higher in the severe stenosis group compared to the mild-to-moderate group (639.8 vs. 536.5). The optimal SII cut-off value was 639.67, yielding a sensitivity of 50.4% and a specificity of 66.0% (area under the curve = 0.601; 95% confidence interval (CI) = 0.535-0.668; p = 0.003). An SII ≥639.67 was associated with a significantly higher prevalence of severe stenosis on crude analysis (PR = 1.427; 95% CI = 1.119-1.819; p = 0.004), and this association remained statistically significant after multivariable adjustment (adjusted PR = 1.295; 95% CI = 1.044-1.605; p = 0.018).

Conclusions

An SII cut-off of 639.67 was independently associated with severe coronary stenosis as defined by the Gensini score, with moderate discriminatory performance. SII may serve as a complementary tool in clinical assessment to prioritize further diagnostic evaluation, particularly in resource-limited settings. However, it should not be used as a standalone diagnostic criterion. Given the cross-sectional design of this study, prospective studies are warranted to validate these findings.

## Introduction

According to the literature, coronary artery disease (CAD) is a pathological condition characterized by the accumulation of atherosclerotic plaque within the coronary arteries [1,2]. A study by Cassar et al. reported that CAD is among the most common causes of death in developing countries, accounting for approximately one in every five deaths [3]. Epidemiological data from 2021 by Ralapanawa et al. identified CAD as the cause of approximately seven million deaths in developing nations [4]. Additionally, CAD imposes a substantial economic burden in both developed and developing countries [5].

In 2019, the European Society of Cardiology (ESC) introduced the term chronic coronary syndrome (CCS) to describe the chronic clinical manifestations of CAD [6]. CCS develops as a consequence of structural or functional alterations within the coronary circulation that produce episodic myocardial ischemia despite the absence of acute plaque rupture [7]. The pathological processes in CCS are progressive and may progress into acute coronary syndrome (ACS). As defined by ESC, CCS encompasses a heterogeneous group of patients, including those with stable ischemic symptoms, previous myocardial infarction or coronary revascularization, suspected ischemic left ventricular dysfunction, vasospastic or microvascular angina, and asymptomatic individuals in whom CAD is detected through screening or follow-up evaluation [6].

Earlier research primarily associated CCS pathophysiology with atherosclerotic stenosis, causing impaired coronary blood flow and resulting ischemia. However, growing evidence highlights the central role of inflammation in CCS. A study by Moore et al. demonstrated that innate immune cells are closely linked to atherosclerosis severity [8]. Atherosclerosis begins with endothelial dysfunction, which stimulates monocyte differentiation into macrophages and dendritic cells that function as antigen-presenting cells for T lymphocytes. The adaptive immune system, particularly T and B cells, produces various inflammatory mediators with both pro-inflammatory and anti-inflammatory properties [9].

In recent years, blood-based laboratory parameters such as the systemic immune-inflammation index (SII) have been developed to assess systemic inflammatory and immune responses [10]. SII is calculated from neutrophil count (cells/μL), platelet count (cells/μL), and lymphocyte count. Elevated SII generally reflects heightened inflammatory or immune activity and has been associated with malignancy, severe infections, and autoimmune disorders [11,12]. Unlike the neutrophil-to-lymphocyte ratio (NLR) or platelet-to-lymphocyte ratio (PLR), which each capture only a single axis of immune dysregulation, the SII simultaneously incorporates neutrophil, platelet, and lymphocyte counts into a single composite index.

Within cardiovascular disease, Xia et al. found that high SII values (>655.56) were associated with increased cardiovascular mortality in the general population, supporting the link between neutrophils, platelets, lymphocytes, and cardiovascular disease progression [11]. Another study by Ilmasari et al. reported that elevated SII (>1,459.59) independently predicted mortality in chronic heart failure [13]. Additionally, a study by Kelesoglu et al. demonstrated that SII serves as an independent predictor of coronary collateral circulation [14].

Interestingly, a study by Liu et al. reported that SII correlates with coronary stenosis severity assessed using the Gensini score, showing strong discriminative capacity (sensitivity = 71%, specificity = 86%) [15]. The score incorporates both the degree of luminal narrowing and the anatomical significance of the affected coronary segments. Therefore, higher scores represent not only more severe stenosis but also a greater overall atherosclerotic burden. Thus, the Gensini scoring system can offer a validated and quantitative method for assessing the anatomical severity of coronary stenosis based on angiography [16].

Connecting SII with the Gensini score is scientifically important because these two parameters capture different but complementary dimensions of CAD pathophysiology. SII reflects systemic inflammatory activation, which plays a central role in atherosclerotic initiation, progression, and plaque destabilization. Meanwhile, the Gensini score, in contrast, provides a direct anatomical quantification of coronary obstruction. Therefore, studying the relationship between SII and the Gensini score allows researchers to determine whether systemic inflammatory status corresponds to the actual anatomic burden of coronary disease. Establishing such a relationship, particularly in an understudied population such as Indonesia, may support the development of SII as a low-cost, readily accessible biomarker capable of estimating coronary disease severity before angiography. This could facilitate earlier risk stratification, more efficient clinical decision-making, and improved prioritization for diagnostic and therapeutic interventions in CCS patients. Thus, we aim to determine the association between SII and the severity of coronary artery stenosis, assessed by the Gensini score, in CCS patients undergoing coronary angiography.

## Materials and methods

Study design

This study used a cross-sectional design utilizing secondary data sources. The research was conducted at Cipto Mangunkusumo National General Hospital. The target population comprised CCS patients who had undergone elective coronary angiography. All coronary angiograms were evaluated together by at least two reviewers to ensure assessment reliability. The accessible population included CCS patients who underwent elective coronary angiography at Cipto Mangunkusumo National General Hospital from January 2023 until the required sample size was achieved, which was December 2025. A consecutive sampling approach was used in this study. Ethical approval was obtained by Cito Mangunkusumo National General Hospital (protocol number: 25-08-1207).

Inclusion and exclusion criteria

Exclusion criteria were patients undergoing coronary angiography for ACS, patients with a history of coronary revascularization, including percutaneous coronary intervention or coronary artery bypass graft surgery, individuals with chronic conditions such as stage 4-5 chronic kidney disease on renal replacement therapy, chronic liver disease (cirrhosis), acute infections (e.g., pneumonia, urinary tract infection), chronic infections (e.g., HIV, pulmonary tuberculosis), or hematologic disorders. Patients with acute non-infectious disease were also excluded. Stage 4-5 chronic kidney disease was defined as a glomerular filtration rate less than 30 mL/minute, in accordance with the Kidney Disease: Improving Global Outcomes classification [17]. Other exclusion criteria were patients with carotid artery disease or peripheral arterial disease, patients diagnosed with malignancy, individuals with autoimmune diseases, or those receiving immunosuppressive therapy such as corticosteroids. Finally, patients with incomplete medical record data were excluded, ensuring that only patients with complete datasets were included in the final analysis.

Statistical analysis

The collected data included baseline characteristics (age, sex, and cardiovascular risk factors such as hypertension, diabetes, dyslipidemia, nutritional status, smoking history, prior stroke, and previous myocardial infarction) and laboratory parameters. Laboratory assessments comprised complete blood counts obtained, along with serum creatinine, lipid profile, and left ventricular ejection fraction measured within the preceding six months. Coronary stenosis severity was quantified using the Gensini scoring system based on angiographic findings [18,19].

Data were gathered using structured research forms, medical record documentation, and angiography reports. All extracted data were organized into a spreadsheet format, coded, and subsequently analyzed using SPSS software (IBM Corp., Armonk, NY, USA). Categorical variables were summarized as proportions, while numerical variables were presented as mean and standard deviation for normally distributed data or median for non-normally distributed data. Normality was assessed using the Kolmogorov-Smirnov test due to the sample size exceeding 50 subjects.

For comparisons of mean SII values across coronary stenosis severity categories, independent t-tests were applied for normally distributed variables, whereas Mann-Whitney tests were used for non-normal distributions. When statistically significant differences were identified, further evaluation included determination of the optimal SII cut-off value along with corresponding sensitivity and specificity using receiver operating characteristic (ROC) analysis, yielding the area under the curve (AUC) with a 95% confidence interval (CI).

Bivariate analysis assessed the association between coronary stenosis severity based on the categorical Gensini score and SII values categorized according to the identified cut-off, using chi-square tests for normally distributed data or Fisher’s exact tests for non-normally distributed data. Multivariate analysis was performed using log-binomial regression, incorporating potential confounders with p <0.250 in the bivariate stage. This approach generated both crude and adjusted prevalence ratios (PRs) with 95% CIs, and variables producing >10% change in the PR were considered confounders.

## Results

The study included 296 patients diagnosed with CCS who underwent elective coronary angiography and met the predetermined inclusion and exclusion criteria. Baseline characteristics of patients are shown in Table 1. The study population had a median age of 59 years and a predominance of individuals aged ≥55 years (70.6%). Males constituted 58.8% of the cohort. Cardiometabolic comorbidities were highly prevalent, including hypertension in 74.0%, diabetes mellitus in 35.1%, and dyslipidemia in 76.0%. The median body mass index (BMI) was 25.1 kg/m², with 53.0% classified as obese, and 36.5% reported a history of smoking. Prior cerebrovascular disease and ACS were identified in 13.2% and 7.8% of subjects, respectively. Use of secondary preventive therapies was common, with 71.6% receiving lipid-lowering agents and 74.0% on antiplatelet therapy.

**Table 1:** Baseline characteristics of patients. BP = blood pressure; BMI = body mass index; ACS = acute coronary syndrome; IQR = interquartile range

Variable	N = 296
Age (years), median (IQR)	59 (53–68)
Age <55 years, n (%)	87 (29.39)
Age ≥55 years, n (%)	209 (70.61)
Male sex, n (%)	174 (58.78)
Female sex, n (%)	122 (41.22)
Systolic BP, median (IQR)	131 (118–140)
Diastolic BP, median (IQR)	78 (70–87.5)
Hypertension, n (%) yes	219 (73.99)
Hypertension, n (%) no	77 (26.01)
Diabetes mellitus, n (%) yes	104 (35.14)
Diabetes mellitus, n (%) no	192 (64.86)
Dyslipidemia, n (%) yes	225 (76.01)
Dyslipidemia, n (%) no	71 (23.99)
BMI, median (IQR)	25.1 (22.3–28.55)
Obesity, n (%) yes	157 (53.04)
Obesity, n (%) no	139 (46.96)
Smoking history, n (%) yes	108 (36.49)
Smoking history, n (%) no	188 (63.51)
Stroke history, n (%) yes	39 (13.18)
Stroke history, n (%) no	257 (86.82)
Prior ACS, n (%) yes	23 (7.77)
Prior ACS, n (%) no	273 (92.23)
Lipid-lowering therapy, n (%) yes	212 (71.62)
Lipid-lowering therapy, n (%) no	84 (28.38)
Antiplatelet therapy, n (%) yes	219 (73.99)
Antiplatelet therapy, n (%) no	77 (26.01)
Ejection fraction (%), median (IQR)	58.55 (38.35-64.35)
Creatinine, median (IQR)	1.0 (0.8–1.28)
Neutrophils (cells/mL), median (IQR)	6090 (5530-6805)
Lymphocytes (cells/mL), mean (SD)	2691 (844)
Platelets (×10³/mL), median (IQR)	252,000 (209,500–306,000)
Gensini score, median (IQR)	13.5 (2.5–72)
Severe stenosis (≥20), n (%)	137 (46.28)
Non-severe stenosis (<20), n (%)	159 (53.72)
Systemic immune-inflammation index, median (IQR)	580.81 (411.48–857.68)

Hemodynamic parameters and laboratory indices were within ranges typical for stable coronary disease: median systolic and diastolic blood pressures were 131 and 78 mmHg, median creatinine was 1.0 mg/dL, and median left ventricular ejection fraction was 58.6%. Median counts for neutrophils, lymphocytes, and platelets were 60.9 × 10³/mL, 26.9 × 10³/mL, and 252 × 10³/mL, respectively, yielding a median SII of 580.81. The median Gensini score was 13.5, and severe angiographic stenosis (Gensini ≥20) was present in 46.3% of patients.

The Mann-Whitney test was used to compare median SII values between severe and non-severe stenosis groups. Results of the Mann-Whitney test showed that patients with severe stenosis had a higher median SII of 639.83 compared with 536.52 in the non-severe group, with a Mann-Whitney U value of 8,685 and a p-value of 0.003 (Table 2).

**Table 2:** Mann-Whitney analysis comparing median SII values between severe and non-severe stenosis groups. IQR = interquartile range

Variable	Stenosis Severity		Mann-Whitney U	P-value
Systemic immune-inflammation index, median (IQR)	Severe (n = 137)	Non-severe (n = 159)	8,685	0.003

Determination of the SII cut-off

ROC analysis demonstrated that SII significantly differentiates severe stenosis, yielding an AUC of 0.601 (95% CI = 0.535-0.668; p = 0.003) (Figure 1). This value reflects low-to-moderate discrimination, although still superior to random chance, indicating that SII alone is insufficient as a standalone diagnostic tool. The optimal cut-off value of 639.67 was derived using the Youden index (0.164), corresponding to a sensitivity of 50.4% and a specificity of 66.0%.

**Figure 1:**
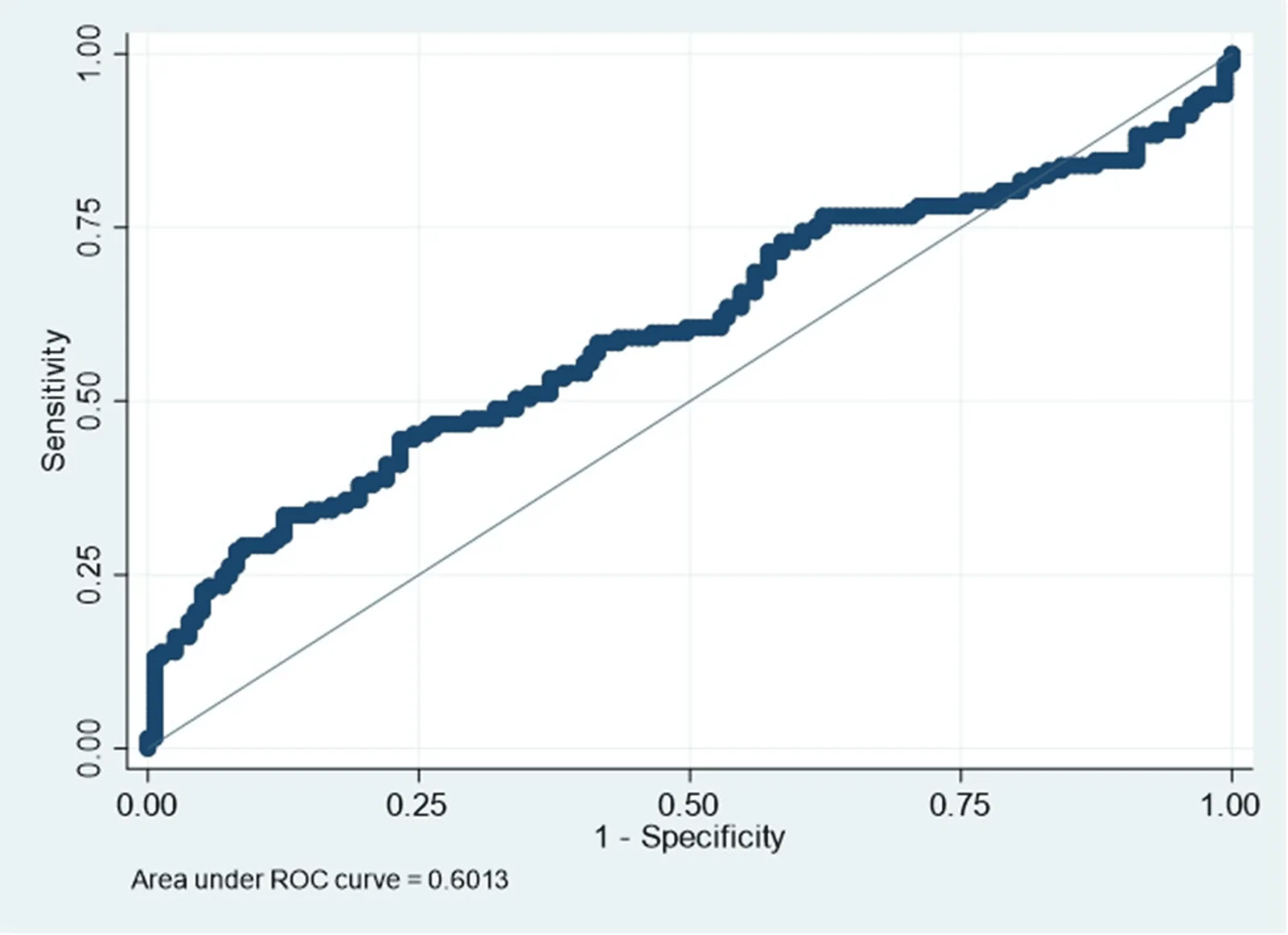
ROC curve of SII for stenosis severity. SII = systemic immune-inflammation index; ROC = receiver operating characteristic

Association between stenosis severity based on the Gensini score and SII values

After determining the SII cut-off through ROC and Youden index analysis (639.67), the continuous SII values were categorized into high SII (≥639.67) and low SII (<639.67). The chi-square analysis demonstrated that the proportion of severe stenosis was higher in the high-SII group compared with the low-SII group (56.10% vs. 39.31%) (Table 3). This association was statistically significant, with a crude PR of 1.427 (95% CI = 1.119-1.819; p = 0.004), indicating that patients with SII ≥639.67 had approximately 43% higher prevalence of severe stenosis compared with those with lower SII values in this study sample (N = 296). These findings confirm a significant relationship between systemic inflammatory status, as reflected by SII, and the severity of coronary stenosis.

**Table 3:** Association between SII categories and coronary stenosis severity based on the Gensini score. n = 296 subjects. SII = systemic immune-inflammation index; CI = confidence interval

SII Category	Stenosis severity		Prevalence ratio (95% CI)	Pearson chi-square	P-value
	Severe (n = 137)	Non-severe (n = 159)	1.427 (95% CI = 1.119–1.819; p = 0.004)	8.16	0.004
≥639.67	69 (56.10%)	54 (43.90%)			
<639.67	68 (39.31%)	105 (60.69%)			

Bivariate analysis of potential confounders and stenosis severity

In the bivariate analysis using the chi-square test, severe stenosis was more frequently observed among males, individuals aged ≥55 years, and patients with diabetes mellitus, dyslipidemia, smoking history, prior ACS, prior stroke, use of antiplatelet therapy, and use of lipid-lowering therapy (Table 4). In contrast, hypertension and obesity did not show statistically significant differences between the severe and non-severe stenosis groups. However, as hypertension is an established risk factor for cardiovascular outcomes, it is included in the multivariate analysis.

**Table 4:** Bivariate analysis based on chi-square tests. ACS = acute coronary syndrome; PR = prevalence ratio; CI = confidence interval

Variable	Severe (n = 137)	Not severe (n = 159)	Chi-square value	PR (95% CI)	P-value
Sex, n (%)					
Male	104 (59.77)	70 (40.23)	30.884	2.209 (1.610–3.032)	<0.0001
Female	33 (27.05)	89 (72.95)			
Age, n (%)					
≥55 years	109 (52.15)	100 (47.85)	9.853	1.620 (1.163–2.259)	0.004
<55 years	28 (32.18)	59 (67.82)			
Hypertension, n (%)					
Yes	105 (47.95)	114 (52.05)	0.935	1.154 (0.855–1.556)	0.349
No	32 (41.56)	45 (58.44)			
Diabetes mellitus, n (%)					
Yes	65 (62.50)	39 (37.50)	16.959	1.667 (1.316–2.110)	<0.0001
No	72 (37.50)	120 (62.50)			
Dyslipidemia, n (%)					
Yes	124 (55.11)	101 (44.89)	29.400	3.009 (1.814–4.993)	<0.0001
No	13 (18.31)	58 (81.69)			
Obesity, n (%)					
Yes	67 (42.68)	90 (57.32)	1.751	0.847 (0.663–1.083)	0.186
No	70 (50.36)	69 (49.64)			
Smoking history, n (%)					
Yes	68 (62.96)	40 (37.04)	19.027	1.716 (1.353–2.175)	<0.0001
No	69 (36.70)	119 (63.30)			
History of previous ACS, n (%)					
Yes	16 (69.57)	7 (30.43)	5.437	1.569 (1.161–2.122)	0.003
No	121 (44.32)	152 (55.68)			
History of stroke, n (%)					
Yes	25 (64.10)	14 (35.90)	5.736	1.471 (1.119–1.933)	0.006
No	112 (43.58)	145 (56.42)			
History of antiplatelet use, n (%)					
Yes	116 (52.97)	103 (47.03)	15.129	1.942 (1.320–2.857)	0.001
No	21 (27.27)	56 (72.73)			
History of antidyslipidemia use, n (%)					
Yes	120 (56.60)	92 (43.40)	32.002	2.796 (1.799–4.349)	<0.001
No	17 (20.24)	67 (79.76)			

Multivariate analysis of independent and confounding variables on stenosis severity

All covariates with p-values <0.250 in the bivariate analysis were included in a log-binomial regression model to examine the stability of the association between high SII (≥639.67) and severe stenosis. High SII remained associated with an increased prevalence of severe stenosis (PR = 1.427; 95% CI = 1.119-1.819; p = 0.004) (Table 5). After sequential adjustment for dyslipidemia, use of lipid-lowering therapy, sex, use of antiplatelet therapy, smoking history, diabetes mellitus, age, prior ACS, prior stroke, obesity, and hypertension, the effect estimate persisted (adjusted PR = 1.295; 95% CI = 1.044-1.605; p = 0.018).

**Table 5:** Multivariate analysis using log-binomial regression model. ACS = acute coronary syndrome; PR = prevalence ratio; CI = confidence interval

Variable	PR (95% CI)	P-value	Δ PR (%)
Crude: systemic immune-inflammation index	1.427 (1.119–1.819)	0.004	—
Adjusted			
Dyslipidemia	1.403 (1.115–1.768)	0.004	1.7
History of antidyslipidemia medication use	1.388 (1.103–1.747)	0.005	3.8
Sex	1.385 (1.113–1.724)	0.003	0.22
History of antiplatelet medication use	1.366 (1.098–1.698)	0.005	1.39
Smoking history	1.338 (1.0758–1.666)	0.009	2.09
Diabetes mellitus	1.308 (1.055–1.624)	0.015	2.29
Age	1.315 (1.059–1.632)	0.003	0.53
Prior ACS history	1.304 (1.051–1.619)	0.016	0.84
Stroke history	1.309 (1.055–1.623)	0.014	0.77
Obesity	1.302 (1.051–1.613)	0.015	0.53
Hypertension	1.295 (1.044–1.605)	0.018	0.54

Assessment of ΔPR across each adjusted model showed only minimal changes (≤2.29%, with the largest shift following adjustment for diabetes mellitus), indicating the absence of meaningful confounding based on the predefined threshold (ΔPR >10%). These findings support SII as an independently associated factor capable of distinguishing severe stenosis within the study population.

## Discussion

This study examined the association between the SII and the severity of coronary artery stenosis, as quantified by the Gensini score, in patients with CCS undergoing coronary angiography. Across 296 patients, nearly half exhibited severe stenosis, and their median SII values were significantly higher than those with non-severe stenosis.

The Mann-Whitney analysis demonstrated a significant difference in SII distributions (median = 639.83 vs. 536.52; p = 0.003), indicating that elevated SII correlates with greater stenotic burden. The ROC analysis yielded an AUC of 0.601 (p = 0.003), reflecting moderate discriminatory capacity and identifying an optimal cut-off of 639.67 (sensitivity = 50.4%; specificity = 66.0%). When this cut-off was applied, individuals with SII ≥639.67 had a 42% higher prevalence of severe stenosis (PR = 1.427; 95% CI = 1.119-1.819; p = 0.004), and this association persisted after multivariable adjustment (adjusted PR = 1.295; 95% CI = 1.044-1.605; p = 0.018). No covariate met the criteria for confounding (>10% change in PR), reinforcing the independent contribution of SII to anatomic coronary disease severity.

These results underscore the biological relevance of SII as a composite inflammatory biomarker incorporating neutrophils, lymphocytes, and platelets. The higher SII values among patients with severe stenosis are consistent with the established pathophysiological links between systemic inflammation and atherosclerotic remodeling [20]. Neutrophils contribute to plaque development by promoting endothelial dysfunction, releasing reactive oxygen species, and neutrophil extracellular traps (NETs), which amplify vascular inflammation and facilitate plaque progression [21]. Platelets further potentiate atherosclerosis through interactions with leukocytes and the vascular endothelium, secretion of pro-inflammatory mediators, and thrombus formation following plaque disruption [22].

The demographic distribution aligns with established CCS epidemiology [23,24]. Most subjects were male (58.78%) and older adults (median age = 59 years). Previous studies from Turkey, China, and Indonesia similarly reported a predominance of older male patients with CCS [15,25]. The high prevalence of comorbidities of hypertension (73.99%), dyslipidemia (76.01%), and obesity (53.04%) also mirrors global CCS cohorts. While the majority did not have diabetes mellitus (64.86%), diabetes showed a significant association with severe stenosis, consistent with its known role in accelerating endothelial dysfunction, oxidative stress, and advanced glycation pathways contributing to atheroma progression.

A considerable proportion of patients had no history of smoking (63.51%), stroke (86.82%), or prior ACS (92.23%). The low prevalence of prior stroke is likely influenced by the predominance of non-severe stenosis in this cohort, as ischemic stroke shares a similar atherosclerotic pathophysiology with CAD. Evidence from ischemic stroke populations shows coronary stenosis in up to 70% of affected individuals, even in the absence of past ACS.

Secondary prevention therapy in this study was high, particularly antiplatelet (74%) and lipid-lowering agents (71.6%), reflecting adherence to guideline-based management. Lipid parameters indicated a cardiometabolic risk profile typical for CCS, with median low-density lipoprotein (LDL) levels of 114.5 mg/dL and preserved ejection fraction averages. The median Gensini score was 13.5, lower than values reported in several Chinese and Indonesian cohorts, reflecting heterogeneity in population risk factors and disease presentation. The median SII in this study (580.81) was broadly comparable to values reported internationally in CCS and severe stenosis populations, reinforcing the external consistency of the inflammatory profile observed.

Age demonstrated a significant association with severe stenosis, consistent with extensive epidemiological evidence linking advancing age with coronary atherosclerotic burden [26]. Structural vascular stiffening, endothelial senescence, immunosenescence, and cumulative oxidative stress collectively contribute to plaque growth and luminal narrowing. Prior studies using varying age cut-offs have uniformly shown increased stenotic risk with increasing age.

Male sex was also significantly associated with severe stenosis (PR = 2.209; p < 0.001), reflecting the absence of estrogen-related vascular protection seen in premenopausal women. Prior studies have shown similar associations, although some cohorts of elderly patients have demonstrated converging risk between sexes due to the loss of hormonal protection in postmenopausal women.

Hypertension did not show a statistically significant association with stenosis severity in this cohort, although the direction of the association suggested increased risk. This finding is consistent with several studies in Asia and Indonesia showing inconsistent relationships between hypertension and Gensini-defined stenosis severity. Variability may be attributable to differing hypertension durations, treatment adequacy, and measurement definitions across studies.

In contrast, diabetes mellitus demonstrated a strong and significant association with severe stenosis, consistent with evidence that hyperglycemia drives endothelial damage, inflammatory activation, and lipid deposition. Dyslipidemia exhibited an even stronger association (PR = 3.009; p < 0.001), consistent with its central role in lipid-rich core formation and plaque progression. These findings align with studies demonstrating dyslipidemia as an independent determinant of stenotic burden. Diabetes is also characterized by persistent low-grade systemic inflammation, which contributes to elevated neutrophil and platelet counts and relative lymphopenia, thereby increasing SII values [27]. Consequently, SII may partially capture the inflammatory milieu associated with diabetes, reflecting the close interplay between metabolic dysregulation and vascular inflammation.

Obesity did not show a significant positive association in this study. This might be due to the “obesity paradox” described in cardiovascular disease, where BMI-based obesity appears protective due to its inability to accurately capture visceral or pericardial adiposity [28]. Evidence indicates that pericardial fat is a superior predictor of coronary stenosis severity compared with BMI [28]. Thus, the value of BMI may be limited in predicting stenosis.

Smoking was strongly associated with severe stenosis (PR = 1.716; p < 0.001), consistent with established pathways linking tobacco exposure to endothelial dysfunction, impaired nitric oxide signaling, oxidative stress, and enhanced plaque formation. A history of stroke was also significantly associated with severe stenosis, reflecting shared atherosclerotic mechanisms across vascular territories. In addition, a history of ACS significantly increased the probability of severe anatomic stenosis, consistent with the presence of complex or unstable plaques and multivessel disease in patients with prior ACS events.

Use of antiplatelet and lipid-lowering therapies was associated with severe stenosis. While this may initially appear counterintuitive, it likely represents reverse causality where clinicians preferentially prescribe these therapies to patients with more pronounced CAD or higher risk features. Thus, patients with established CAD or higher estimated cardiovascular risk are more likely to receive these therapies in routine clinical practice. Furthermore, it is likely that patients receiving antiplatelet and lipid-lowering therapies had higher disease burden. Consequently, adjustment may have resulted in partial overadjustment or collider bias, potentially attenuating the associations observed in the multivariable analysis. These findings should therefore be interpreted as reflecting underlying disease severity rather than the effects of antiplatelet or lipid-lowering therapy themselves.

The optimal cut-off of 639.67 derived from the ROC analysis of this study yielded moderate discriminatory performance (AUC = 0.601). Thus, SII demonstrated an independent association with stenosis severity but lacked sufficient accuracy for risk stratification as a sole predictor. Instead, SII may serve as an accessible, inexpensive adjunct to refine cardiovascular risk assessment in CCS. As SII contains accessible biomarkers, it may be used readily in clinical settings. Nevertheless, SII should be compared with other cardiovascular risk factor scoring systems to determine the effectiveness of SII.

Given its biological relevance in integrating neutrophil-mediated inflammation, lymphocyte suppression, and platelet activation, SII offers a mechanistic reflection of systemic inflammatory tone. This reinforces the continuum connecting inflammation to the anatomical burden of CAD. However, the moderate AUC underscores that atherosclerosis is multifactorial and cannot be sufficiently characterized by inflammatory indices alone. Future studies should investigate whether SII provides incremental prognostic value when combined with established clinical risk scores, imaging modalities, and other inflammatory biomarkers such as high-sensitivity C-reactive protein, NLR, or PLR.

This study has several limitations. First, its cross-sectional design precludes establishing temporal or causal relationships between elevated SII and the severity of coronary artery stenosis. Although a significant association was observed, it cannot be determined whether increased systemic inflammation preceded or resulted from more advanced atherosclerotic disease. Second, SII was dichotomized using the optimal cut-off derived from the Youden index to facilitate clinical interpretation and risk stratification. However, because SII is inherently a continuous variable, this approach may have reduced statistical power. Another limitation is that the time to contrast was not measured in this study, as the time for contrast to pass from the left coronary ostium to the coronary sinus can be associated with CCS [29]. Finally, lipid profile measurements, particularly LDL-cholesterol levels, were unavailable for a substantial proportion of patients due to incomplete medical records. Prospective longitudinal studies with comprehensive lipid profiling and analyses treating SII as a continuous variable are warranted to further clarify the clinical utility of SII in CCS.

## Conclusions

This study demonstrates that the SII is independently associated with the severity of coronary artery stenosis in patients with CCS. Higher SII values were associated with higher Gensini scores in this study, and an SII cut-off of 639.67 provides small discriminatory capability. Although SII alone is insufficient for definitive risk stratification, it constitutes a biologically relevant and statistically consistent adjunctive marker that enhances clinical assessment of inflammatory activity and coronary disease severity. Given its low cost, accessibility, and mechanistic alignment with atherosclerotic progression, SII may play a supportive role in the multimodal evaluation of coronary risk. Due to the moderate discriminatory performance of SII, future investigations should evaluate the prognostic value of SII in predicting clinical outcomes compared with other scoring systems and its potential integration into standardized risk stratification tools.
